# Our Penal Machinery and Its Victims

**Published:** 1887-01

**Authors:** 


Our Penal Machinery and Its Victims. *By* John P. Altgeld. *New and Revised Edition. Octavo, pp. 151. Chicago:* A. C. McClurg & Company.

Too often those who approach the subject of crime, and the care of criminals, whether from the humanitarian or sociological standpoint, are termed visionaries or doctrinaires, their views are scouted, and some lawyer whose legal acumen does not extend beyond caring what the law is, providing only that it is fixed, pronounces them impractical. To all thinking minds this indictment of our penal machinery must come with added force from the pen of an accomplished jurist, now occupying a seat on the bench.

We can mention but briefly a few of the many facts gathered into this little volume, which might easily have been expanded to greater bulk.

After a brief description of the size and cost of our penal machinery, he is compelled to admit that " it does not deter the young offender, and it seems not to reform nor restrain the old offender. This being so, one is naturally led to ask whether there is not something wrong with the system; whether it is not based on a mistaken principle; whether it is not a great mill which, somehow or other, supplies its own grist; a maelstrom which draws from the outside and then keeps its victims moving in a circle until swallowed in the vortex."

The broad principle of the law is laid down that society never claimed the right of punishment for violation of the moral law, but only for infraction of its own laws. Therefore, anything which is done that is not necessary for the protection of society is unwarranted and wrong. The present system is characterized as "formal, iron-bound and superficial." Not content with pointing out defects, some remedies are suggested. The abandonment of the present idea of *punishment* and the substitution of protection of society as the sole object to be attained in dealing with offenders. The subject of "indeterminate sentence" is very fully discussed.

The work is broad and philosophical, and will, we trust, do much to further the greatly-needed reform in the administration of criminal law. The numbering of so good a lawyer among the "doctrinaires" presages much for the ultimate triumph of a scientific criminal jurisprudence. H. N. M.

